# The In Vitro Anti-Pseudomonal Activity of Cu^2+^, Strawberry Furanone, Gentamicin, and Lytic Phages Alone and in Combination: Pros and Cons

**DOI:** 10.3390/ijms22189830

**Published:** 2021-09-11

**Authors:** Agata Dorotkiewicz-Jach, Pawel Markwitz, Zuzanna Drulis-Kawa

**Affiliations:** Department of Pathogen Biology and Immunology, Institute of Genetics and Microbiology, University of Wroclaw, Przybyszewskiego 63/77, 51-148 Wroclaw, Poland; agata.dorotkiewicz-jach@uwr.edu.pl (A.D.-J.); pawel.markwitz2@uwr.edu.pl (P.M.)

**Keywords:** *Pseudomonas aeruginosa*, 4-hydroxy-2,5-dimethyl-3(2H)-furanone, copper, gentamicin, lytic phages, combined therapy, anti-virulent activity

## Abstract

In this study, we investigated the anti-pseudomonal activity of cupric ions (Cu^2+^), strawberry furanone (HDMF), gentamicin (GE), and three lytic *Pseudomonas aeruginosa* bacteriophages (KT28, KTN4, LUZ19), separately and in combination. HDMF showed an anti-virulent effect but only when applied with Cu^2+^ or GE. GE, at a sub-minimal inhibitory concentration, slowed down phage progeny production due to protein synthesis inhibition. Cu^2+^ significantly reduced both the bacterial cell count and the number of infective phage particles, likely due to its genotoxicity or protein inactivation and cell membrane disruption effects. Furthermore, Cu^2+^‘s probable sequestration by phage particles led to the reduction of free toxic metal ions available in the solution. An additive antibacterial effect was only observed for the combination of GE and Cu^2+^, potentially due to enhanced ROS production or to outer membrane permeabilization. This study indicates that possible interference between antibacterial agents needs to be carefully investigated for the preparation of effective therapeutic cocktails.

## 1. Introduction

Broad usage of antibacterials in medicine, agriculture, and everyday life (e.g., household disinfectants) brought us to the era of omnipresent bacterial multidrug resistance. Currently, we are facing an urgent need for new treatment options against most life-threatening pathogens, especially from the ESKAPE group [1,2]. Among them, *Pseudomonas aeruginosa* is of great concern due to its high antibiotic resistance and fast adaptability to harsh environmental conditions (low nutrient availability, toxic compound presence, low oxygen level). This pathogen produces a broad panel of virulence factors enabling efficient host invasion and colonization, and additionally, the ability to form biofilms gives auxiliary protection against immune responses and other unfavourable conditions. Complex mechanisms, including the two-component systems (TCS), and secondary messengers involved in Quorum Sensing (QS) and c-di-GMP (diguanylate cyclase, DGC) networks, are responsible for *P. aeruginosa* environmental fitness and control of the expression of virulence factors [3]. The intrinsic, adaptive, and acquired drug resistance mechanisms make *P. aeruginosa* a serious clinical problem, especially in the immunocompromised Cystic Fibrosis (CF) sufferers and burn unit patients [4]. Nevertheless, commonly used antibiotics, such as gentamicin (aminoglycoside), are still potent treatment options in pseudomonal infections. The gentamicin mode of action impairs protein synthesis and at high concentrations also disrupts the cytoplasmic membrane [5,6].

A great variety of modern and innovative antipseudomonal approaches have also been investigated as options including quorum sensing inhibitors, antimicrobial peptides, and phages [7,8,9]. The most promising turned out to be the antibiotic/phages therapy as lytic phages can control drug resistance emergence due to fast bacterial count reduction [10,11,12].

Halogenated furanones produced by seaweed *Delisea pulchra* were proposed as anti-virulent molecules overriding communication signals implied in QS mechanisms and their combination with aminoglycosides increased bacterial drug susceptibility [13]. Since that discovery, a wide panel of furanone molecules has already been tested as a potential alternative or supportive antimicrobial option [14,15]. One of the examples is the 4-hydroxy-2,5-dimethyl-3(2H)-furanone (HDMF, strawberry furanone), a natural and nonhalogenated molecule found in fruits. It was shown that among four different *P. aeruginosa* QS systems, two (Rhl and Las) are under the control of acyl-homoserine lactone (AHL) signals which are competing with HDMF molecules for receptors [7,16]. The application of HDMF reduced the biofilm formation (42.6%), LasA protease (53.8%), rhamnolipid (40.9%), and pyocyanin (51.4%) production in *P. aeruginosa* PAO1 [16].

The antimicrobial activity of metals such as copper and its ions are well known, therefore, are applied in constructions, water systems, agriculture, and medicine [17,18,19,20]. Cu^2+^ ions are prone to bind to inorganic and organic ligands forming stable complexes with -NH_2_, -SH, and, -OH groups [21]. The unspecific cupric ions’ mode of action is related to hydrogen peroxide (H_2_O_2_) and reactive oxygen species (ROS) generation [22]. The damage of crucial molecules such as DNA, proteins, and phospholipids is also reported [6,23,24]. Heavy metals and oxidative stresses are stimuli for QS response, mainly through Las and Rhl systems [25]. Both QS systems act in the hierarchy and are involved in the expression of different virulence determinants [26]. Moreover, Cu^2+^ can induce antibiotic resistance and virulence in *P. aeruginosa* [27,28,29,30]. In that context, the application of copper as an antibacterial surface coating or additive to everyday items might be of some concern.

Since non-traditional antibacterials to combat pathogenic bacteria are more widely investigated we wanted to verify the activity of the aforementioned agents when applied in cocktails to discover any mutual beneficial or adverse interactions. To accomplish the study, we used *P. aeruginosa* PAO1 as a model strain to test the activity of cupric ions (Cu^2+^), HDMF, gentamicin (GE), and three *Pseudomonas* lytic phages, alone and in combination. We were interested in finding out whether the different modes of action of each agent might influence the overall antibacterial or anti-virulent effect. As *P. aeruginosa* has complex mechanisms of metabolism regulation, we monitored the bacterial growth rate and selected a virulence determinant production (elastase, pyocyanin, and pyoverdine). Elastase (a proteolytic enzyme [31]) and pyocyanin (a phenazine toxin [32]) are produced under the Las and Rhl (QS) control [25,33], thus, we expected to see an inhibitory impact of HDMF. The third analysed was pyoverdine, a complex fluorescent siderophore synthesised in a ribosomal-independent pathway, involved in iron uptake and a chelator of heavy metals [26,34,35,36,37,38], therefore, interacting with cupric ions tested in this study and possibly insensitive to protein production inhibition caused by GE.

## 2. Results

The first part of the study was focused on the antibacterial and anti-virulent activity of all chemicals separately and in combination, whereas the second was dedicated to the lytic activity of phages mixed with the above chemicals.

### 2.1. Cu^2+^ in Combination with GE Show Additive Antibacterial Effect, Whereas HDMF Neutralizes the Copper-Induced Virulence and Toxicity

**Figure 1:**
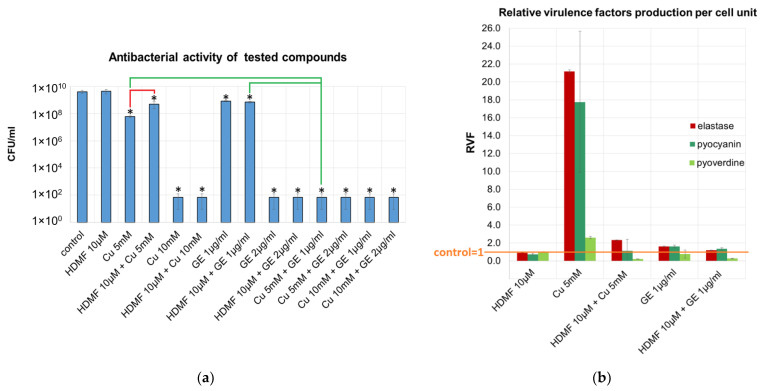
The impact of Cu^2+^, HDMF, and GE on *P. aeruginosa* PAO1 growth rate and virulence determinant production: (**a**) colony count after 20 h of incubation; (**b**) relative virulence factor production (RVF) per cell unit compared to the untreated control; *statistically significant differences according to PAO1 control (*p* < 0.05), brackets indicate statistically significant differences between agent combinations (*p* < 0.05).

We tested the antibacterial activity of Cu^2+^ and HDMF in combination with GE by measuring the bacterial growth rate and production of three virulence factors. The effect of all tested concentrations and combination on the colony count reduction is provided in Supplementary Materials Figure S1, whereas the results of selected agents on bacterial growth and the production of virulence factors is presented in Figure 1. The application of HDMF at 10 µM, as reported previously [16,39], had no adverse effect on the *P. aeruginosa* PAO1 growth rate. The 10 mM of Cu(NO_3_)_2_ (Cu 10 mM) reduced the bacterial count up to 7 logs after 20 h of incubation therefore was assigned as the bactericidal concentration. Although Cu 5 mM did significantly lower the colony count compared to the untreated control, the culture growth was still noticeable (6 × 10^7^ CFU/mL), therefore, it was set as a sub-inhibitory concentration of cupric ions (sub-MIC). Similarly, the GE at 1 μg/mL were assessed as sub-MICs whereas 2 µg/mL as MIC against *P. aeruginosa* PAO1 (Figure 1a). It turned out that the combination of Cu 5 mM with antibiotic at 1 μg/mL gave an additive effect with a fractional inhibitory concentration index (FIC_I_) of equal 1 compared to each agent individually as the bacterial count dropped below 10^2^ CFU/mL (Figure 1a, green bracket). Interestingly, HDMF significantly worsened the antibacterial activity of Cu 5 mM (*p* > 0.05) with the final value of 1 × 10^9^ CFU/mL compared to 6 × 10^7^ CFU/mL for Cu 5 mM alone (Figure 1a, red bracket). The application of HDMF at 10 µM had no adverse effect on GE activity.

The purpose of our experiments was to look for the enhanced antibacterial and anti-virulent effect of tested combinations (Figure 1b and Figure S2). Therefore, the samples containing Cu 10 mM, GE 2 µg/mL, and Cu 5 mM + GE 1 μg/mL, which are recognized as bactericidal, were excluded from those analyses. Direct measurement of all tested virulence determinants revealed a lack of HDMF impact according to the untreated control when utilized alone. Likewise, no statistically significant differences were seen for the HDMF combination with Cu^2+^ nor GE (Figure S2, Table S1) according to both single molecules with an exception for elastase production when mixed with 1 μg/mL GE. The remaining agents/cocktails significantly reduced all virulence markers according to the untreated control.

As the direct measurement of virulence determinant production does not take into account the differences in the final CFU/mL (Figure 1a), the detected levels have been recalculated per cell unit and expressed as a relative virulence factor production (RVF, Figure 1e), where the control sample means RVF = 1.

The conversion to RVF revealed that Cu 5 mM alone increased the RVF of QS-regulated elastase and pyocyanin by 21.1 folds and 17.7 folds, respectively, whereas, for pyoverdine it was by 2.6 folds according to the untreated control. That effect was dramatically impaired by the addition of HDMF with the final RVF values of 2.3, 1, and 0.2 for elastase, pyocyanin, and pyoverdine, respectively. In the presence of GE 1 µg/mL, the RVF of produced virulence determinants varied between 0.5 and 1.5, nevertheless, the combination with HDMF at least reduced the RVF of pyoverdine.

**Figure 2:**
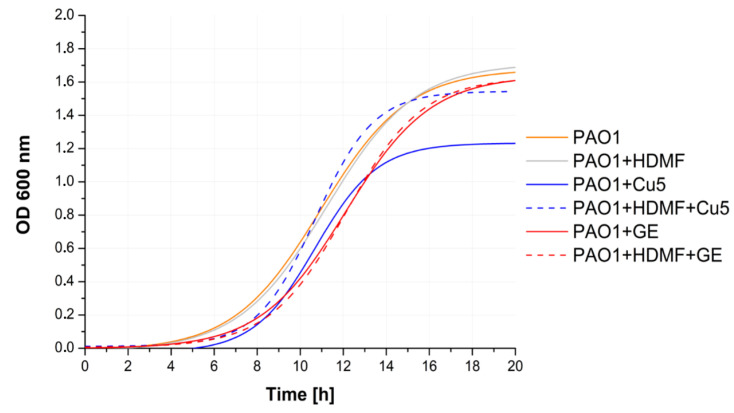
The impact of tested chemicals and their combination on the *P. aeruginosa* PAO1 growth kinetics. Fitted curves were established on the average of triplicate OD_600_ measurement and the SD in the range +/−0–0.3. The kinetics were measured at 20 min intervals).

To explain RVF differences between the tested combinations bacterial growth kinetics was performed (Figure 2). The HDMF, and GE alone or in combination, did not change the growth kinetics, although, it reduced some RVF levels calculated after 20 h of incubation suggesting that bacteria maintained at least the basic metabolic activity crucial for growth.

The Cu 5 mM treatment (solid blue curve) reduced the final OD_600_ compared to the control confirming a sub-MIC effect shown in Figure 1a. Considering the RVF calculation (Figure 1b), it seemed that Cu 5 mM reduced bacterial propagation while simultaneously forcing cells to overproduce virulence factors. In that context, the addition of HDMF neutralized the Cu 5 mM toxic effect (dash blue curve) inhibiting virulence determinant production as the observed growth, and RVF levels were almost identical to the untreated PAO1 control.

### 2.2. Cu^2+^ and HDMF Do Not Enhance the Drug Resistance Emergence when Applied for 20 h

Copper and antibiotics can stimulate bacteria to develop drug resistance, therefore, the impact of tested agents at sub-MIC concentrations against *P. aeruginosa* PAO1 was investigated. Bacteria were incubated with HDMF 10 µM, Cu 5 mM, GE 0.5 µg/mL, and their combinations HDMF + GE 0.5 µg/mL, HDMF + Cu 5 mM, Cu 5 mM + GE 0.5 µg/mL for 20 h, and the antibiotic susceptibility was then determined. We have chosen GE at 0.5 µg/mL as its combination with Cu 5 mM did not eradicate bacteria conversely to the 1 µg/mL of GE (Figure S1). The untreated PAO1 control was susceptible to all applied antibiotics such as cefotaxime, ceftazidime, piperacillin, piperacillin/tazobactam, imipenem, gentamicin, amikacin, and ciprofloxacin (Figure S2) and none of the tested chemicals nor their combinations affected the drug sensitivity patterns. Differences in inhibition zone diameters varied up to ±2 mm according to the untreated bacteria and did not change the interpretation of drug susceptibility according to EUCAST breakpoints (https://eucast.org/ Version 8.0, accessed on 1 January 2020).

### 2.3. Cu^2+^ and GE Reduce the Effective Propagation of Lytic Phages Whilst HDMF Is Neutral

**Figure 3:**
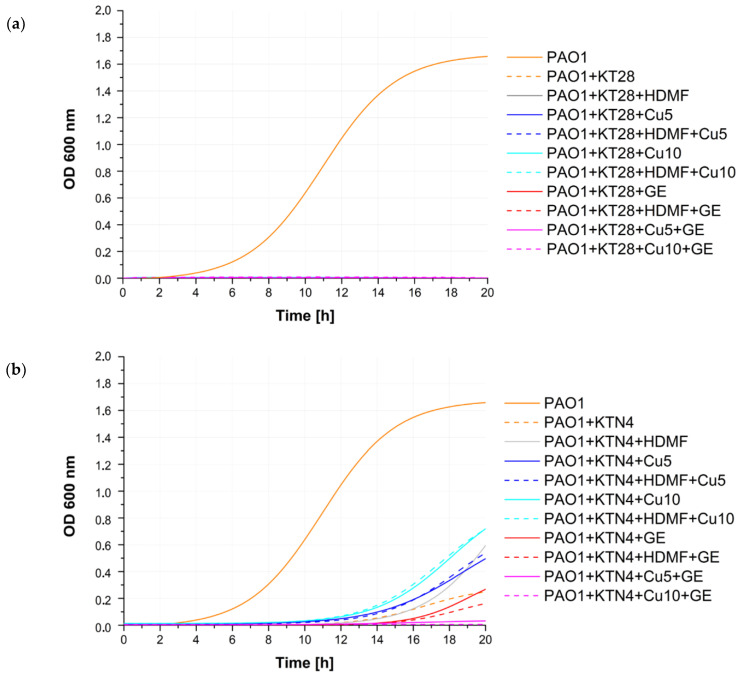

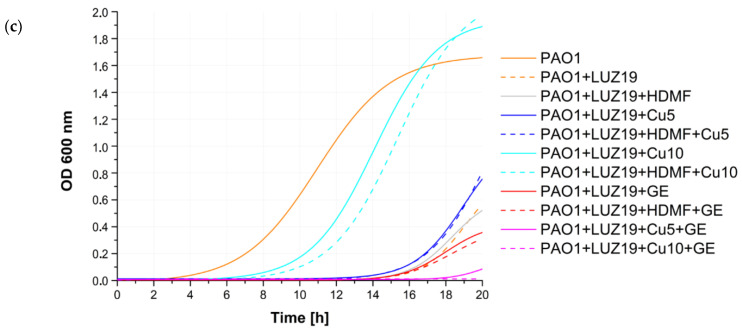
The impact of Cu^2+^, GE, and HDMF and their combination on the *P. aeruginosa* PAO1 culture growth when infected with (**a**) KT28, (**b**) KTN4, or (**c**) LUZ19 phages. The control consisted of untreated culture (orange solid curve). Fitted curves were established on the average of triplicate OD_600_ measurement and the SD in the range +/−0–0.3. The kinetics were measured at 20 min intervals.

**Figure 4:**
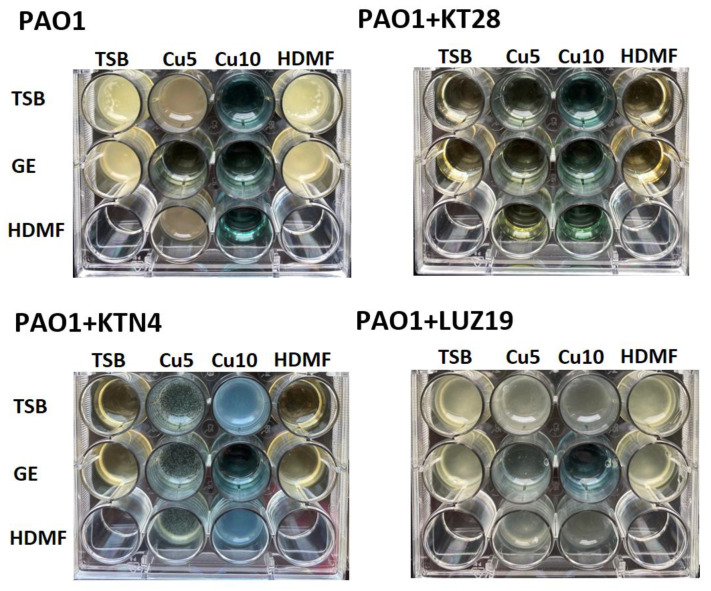
The image of *P. aeruginosa* PAO1 culture (20 h) treated with Cu^2+^, GE, HDMF and their combination when infected with KT28, KTN4, or LUZ19 phages.

Principles of phage experiments are slightly different from those of drugs, mainly due to the propagation lifestyle of phages. To make the study more comprehensive, three lytic *Pseudomonas* representative phages (KT28, KTN4, and LUZ19) were selected, each belonging to a different morphology group (myovirus, giant virus, and podovirus, respectively) and recognizing different receptors, lipopolysaccharide (LPS) or type IV pilus (T4P). Moreover, the phage LUZ19 is equipped with the exopolysaccharide-degrading enzyme able to digest the LPS/biofilm matrix. To compare the impact of Cu^2+^, GE, and HDMF on phage propagation, different experimental procedures were implemented. Firstly, we tested the *P. aeruginosa* PAO1 growth impairment in the presence of KT28, KTN4, and LUZ19 phages at the multiplicity of infection of 10 (MOI = 10) (Figure 3 and Figure 4).

Growth curves revealed a strong antibacterial activity of phages used separately and with the combination of Cu^2+^ and GE in all cases (magenta solid and dash curves), where overlapping effects of Cu^2+^ toxicity, GE protein biosynthesis inhibition, and lytic activity of phages were present. The rest of the tested agents and their combinations gave various results in a phage-dependent manner. Phage KT28, alone and in combination with all tested chemicals, efficiently inhibited the *P. aeruginosa* population growth for 20 h of incubation time (Figure 3a and Figure 4). For bacteria infected with the giant phage KTN4, visible growth was observed after 12 h when Cu 5 mM (blue solid curve), Cu 10 mM (cyan solid curve), and their combination with HDMF (dash curve respectively) were added (Figure 3b and Figure 4). Interestingly, Cu 10 mM and its combination with HDMF, gave the dense surface growth of bacterial culture (Figure 4), whereas, for Cu 5 mM, single colonies were visible in the culture. Additionally, bacteria were also able to grow when infected with phage KTN4 itself and in combination with HDMF suggesting the emergence of phage-resistant clones. The addition of GE inhibited bacterial growth similarly to the phage itself. A similar observation was made for phage LUZ19 infection where the population density started growing in the presence of Cu 10 mM and Cu 10 mM + HDMF after 8 h of experiment. In the case of phage LUZ19/Cu5/HDMF/Cu 5 mM combinations, the growth rate was strongly reduced up to 14 h of incubation (Figure 3c). Observed bacterial growth in the presence of Cu 10 mM combined with phage KTN4 or phage LUZ19 was thought-provoking and disposed us to check the final phage titre after 20 h of propagation in the presence of bacteria and single tested chemicals (Table 1).

In the presence of HDMF or Cu 5 mM, all phages were able to propagate to the same final titres as in the control. The presence of GE 1 µg/mL reduced the final phage titre of all phages. Phage propagation could have been impaired due to the bacteriostatic activity of GE slowing down the bacterial protein synthesis. Interestingly, in the presence of bactericidal Cu 10 mM, the phage particle count was decreased by 2 or 5 logs for KT28 or KTN4 and LUZ19 phages, respectively, although the bacterial growth was seen especially for phage LUZ19 infection (Figure 3). Therefore, the PAO1 population growth dynamics were startling when treated with the phage/Cu combination (Figure 3 and Figure 4). To verify whether the phage titre is dependent on the viability of bacterial culture or the anti-phage action of copper ions, the phage neutralization experiments were then carried out in the absence of bacteria.

### 2.4. Cu^2+^ Reduce the Number of Infective Phage Particles Whilst HDMF and GE Are Neutral

The influence of Cu^2+^, HDMF, and GE as single chemicals and in combination on phage particles infectivity was examined after 4 h of incubation without bacteria (Table 2).

Obtained results allowed us to divide the tested agents into two groups: phage neutral (GE and HDMF) and PFU reducing agents (Cu). Cupric ions at both concentrations (5 mM and 10 mM), as well as in combination with HDMF or GE, reduced the phage titre significantly. The siphovirus KT28 was only susceptible to Cu 10 mM (2 logs reduction). Giant phage KTN4, as well as podovirus LUZ19, were more prone to cupric ion toxic activity with a drop of 2 logs and 5 logs of PFU/mL when incubated with Cu 5 mM and Cu 10 mM, respectively. Observations with phage titre reductions were consistent with the previously performed *P. aeruginosa* PAO1 growth in the presence of phages (Figure 3) and phage propagation (Table 1). Cu-insensitive phage KT28 was able to propagate in and kill the *P. aeruginosa* population. The addition of Cu 10 mM caused partial phage particles neutralization, but a high concentration of Cu^2+^ itself and viable phage particles, were still able to eradicate bacteria, thus, no visible growth was detected (Figure 4). In contrast, bacterial growth was observed after 20 h of incubation when giant KTN4 and podovirus LUZ19 infection was combined with cupric ions (Figure 3 and Figure 4). That could mean probable Cu^2+^ sequestration on phage particles resulting in mutual neutralization of the Cu/phage cocktail.

### 2.5. Cu^2+^ Irreversibly Inactivate Phage Infectivity but Does Not Disturb the Virion Morphology

**Figure 5:**
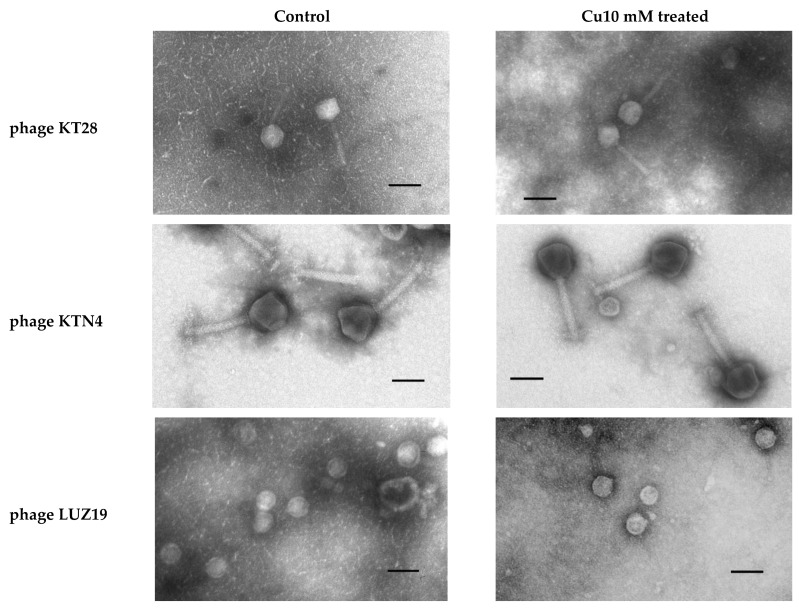
TEM electrographs of phages after 4 h incubation with Cu 10 mM. Magnification 22,000×. Bars indicate 100 nm.

The observed partial or complete phage inactivation in the presence of Cu ions led us to question the toxicity mechanism of the Cu^2+^ action on tested phage particles. The metal ions mode of action responsible for virus inactivation is connected to hydrogen peroxide (H_2_O_2_) and reactive oxygen species (ROS) generation [22], damaging the critical biological molecules such as DNA, proteins, and phospholipids [6,23,24]. First, we checked whether the anti-phage action of Cu^2+^ was a lasting effect. It turned out that the effect of phage titre reduction was stable and even 24-h dialysis was not restored. Therefore, to check cupric ion influence on phage particle stability, we examined the virion morphology using transmission electron microscopy (TEM). As visualized in Figure 5, phage particles were neither destroyed nor deformed compared to the untreated control samples. The shape and size of capsids remained unchanged regardless of the phage-type. Therefore, we concluded that the toxicity of cupric ions should be based on a different mechanism, probably the direct interactions with phage DNA.

## 3. Discussion

The presented study aimed to investigate the impact of Cu^2+^, HDMF, gentamycin, and lytic phages alone and in combination against the model strain of *P. aeruginosa* PAO1. By analysing the results we were able to make several conclusions.

**Figure 6:**
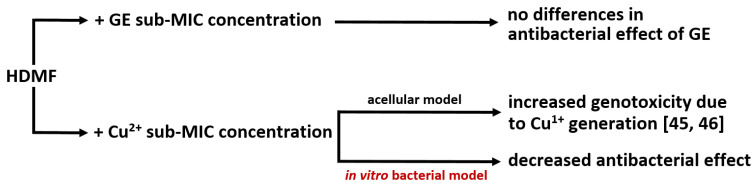
The antibacterial activity of HDMF in combination with GE and Cu^2+^; no enhancement nor decrease in efficacy of GE was observed when combined with HDMF; Cu^2+^ combination with HDMF in an acellular model increase genotoxicity, contrary to the reduced antibacterial effect in in vitro bacterial model (red fonts).

Our first main question was whether the combination of tested chemicals improves the antibacterial effect of single molecules or impair their function. The experiments confirmed that strawberry furanone alone did not inhibit bacterial growth [16] nor induce drug resistance emergence after 20 h of treatment. That is an important feature considering the broad usage of HDMF in everyday life as a safe food additive in beverages, ice cream, and cigarettes, as well as natural organic compounds in fruits [39]. HDMF combined with GE at a sub-inhibitory concentration did not interrupt nor enhance its antibacterial efficacy. To the best of our knowledge, this is the first report discussing the effect of a GE/HDMF mixture. Figure 6 summarises the overall antibacterial activity of HDMF-combined treatment.

The Cu 10 mM application was bactericidal whereas Cu 5 mM significantly limited PAO1 growth, which is consistent with previous reports on CuSO_4_ minimum inhibitory concentration (MIC~10 mM) against planktonic forms of *P. aeruginosa* [40,41]. The combination of HDMF and cupric ions has not been tested yet in in vitro conditions against bacteria, therefore, reduced toxic activity of Cu^2+^ in our experiments shed new insight on HDMF–Cu interactions (Figure 6). This antagonistic effect is even more surprising as HDMF was proven to be a copper-reducing agent initiating the production of superoxide radicals through the reduction of cupric to cuprous ion [42]. In the study by Yamashita et al., superoxide was generated from HDMF in the presence of Cu^2+^ and subsequently, metal-dependent DNA damage was observed. Those experiments were conducted in an acellular model [43] in contrast to a bacterial culture in our case. The observed phenomenon might be related to the probable sequestration or stronger affinity of Cu^2+^ ions to different negatively charged biomolecules such as LPS and other outer membrane constituents which were not present in the Yamashita et al. acellular model.

**Figure 7:**
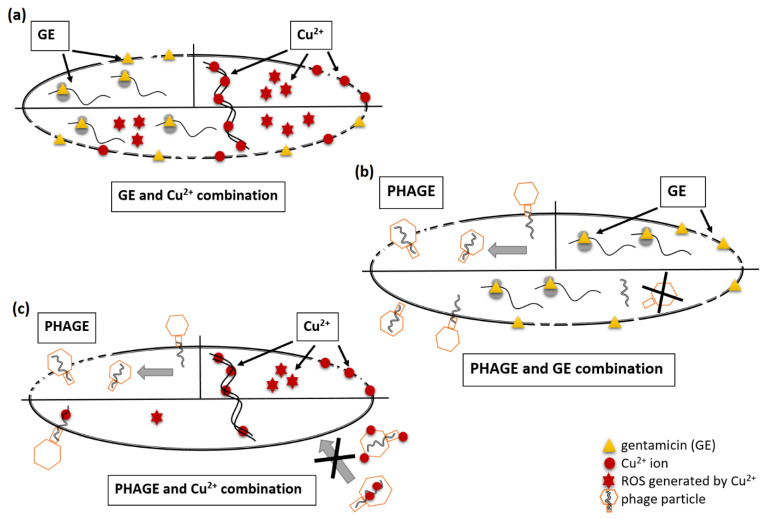
The proposed model of antibacterial action of tested combinations: (**a**) GE + Cu^2+^ combination shows an additive antibacterial effect in enhanced ROS production, outer membrane permeabilization, and bacterial cell lysis; (**b**) simultaneous application of GE and phages results in the protein synthesis inhibition reducing an efficient phage progeny production; (**c**) Cu^2+^ + phages combination causes a mutual adverse effect such as probable cupric ions sequestration by phage particles and the reduction of phage infectivity by the toxic cupric ions. Each panel (**a**–**c**) presents a bacterial cell divided into the top parts representing the mode of action of a particular antibacterial agent applied separately (left and right) and the bottom part of the cell with the proposed activity of combined treatment.

Conversely to HDMF + Cu 5 mM, the Cu 5 mM + GE 1 µg/mL combination significantly improved the antibacterial effect of each molecule alone. That could be explained by the efficient binding of Cu^2+^ ions to the amino groups present in aminoglycosides and the enhanced disruption of the bacterial outer membrane [6,27,44]. Aminoglycosides possess two killing mechanisms, inhibiting the protein synthesis and further damaging the outer membrane saturated with Mg^+2^ and Ca^2+^ ions [5,6] (Figure 7a). Although GE is much larger than the aforementioned ions, it is highly competitive for the negatively charged LPS sites consequently leading to ion displacement and outer membrane perturbance [45]. The copper ions might also replace Mg^+2^ and Ca^2+^ ions increasing the outer membrane perturbance, but the Cu^2+^ mode of action is also related to an increased intracellular ROS level, hydroxyl radical formation, and the impairment of the iron-sulfur dehydratase enzymes [46,47] (Figure 7a). The observed in vitro additive effect (FIC_I_ = 1) may come from the simultaneous activity of both antibacterials elevating the ROS level in the cell (Figure 7a). Unfortunately, considering the therapy, in vivo Cu^2+^-aminoglycoside complex may dramatically change the pharmacodynamics of the drug by strengthening the oxidative reactions resulting in severe side effects [44]. Moreover, the stable Cu^2+^-GE complex cannot be achieved in the blood plasma as histidine replaces GE in those complexes under physiological conditions [48]. Thus, the Cu^2+^-GE composition might only be applied for topical infections, for instance, as an element of modern wound dressings [49,50,51]. Regarding the possible effect of cupric ions on drug resistance emergence [27,52] our data showed no influence of Cu^2+^ sub-MIC concentration after 1 day of *P. aeruginosa* PAO1 selective pressure.

The other aspect of our experiments aimed to investigate the impact of tested chemicals on the lytic activity of *P. aeruginosa* phages (KT28, KTN4, and LUZ19) to verify the potential therapeutic combination in an in vitro model. Although the impact of furanone on lytic phage propagation has not yet been tested, we may assume that furanone affecting the QS signal transduction can eventually modify the expression of bacterial cell surface structures and impact phage adsorption to the targeted receptor [15]. Our data revealed that HDMF at 10 µM did not impair the activity of lytic phages (Figure 7). In the case of the phage combination with GE, the effect can be beneficial, neutral, or negative [53]. Even with a sub-MIC concentration of GE (1 µg/mL) the inhibited protein synthesis influenced an efficient phage progeny production (Table 1, Figure 7b), although, GE itself did not neutralize the phages (Table 2). Our results are in agreement with the previous studies reporting a beneficial treatment combination of phages and streptomycin (aminoglycoside), but only when administrated sequentially [54,55]. Therefore, the mode of antibiotic action should be taken into account while designing phage–antibiotic therapy. Indeed, piperacillin and ceftazidime (β-lactams) blocking the peptidoglycan cross-linking do not disturb phage propagation, and both exhibit a synergistic effect while applied simultaneously with phages [56]. A phage–drug combination is also considered beneficial with the assumption that the drug-resistant portion of the treated population remains sensitive to phage infection [57]. In light of the evolutionary rationale, the simultaneous resistance emergence to antibiotics and phages entails huge metabolic costs, and a slow-growing, less virulent population is easily cleared out by the immune system [53].

The antibacterial Cu^2+^ concentrations also had an adverse effect on viral particles infectivity in a phage-dependent manner (Table 2). The Cu toxicity might be connected to the damage of crucial molecules such as DNA or proteins as reported previously for other viruses [23,24]. As we excluded phage particle damage (TEM analysis), as well as the ROS generation (experiments with bacteria absence), we might, therefore, propose a Cu-DNA intercalation mechanism (Figure 7c). Moreover, Cu^2+^ bind to organic ligands and form stable complexes with -NH_2_, -SH, and -OH groups [21], thus, it may interact with phage virion structural proteins (Figure 7c). This is in line with previous reports describing phage sensitivity to similar concentrations of cupric ions and metallic copper surfaces [58]. Alternatively, the antibacterial activity of cupric ions is neutralized by phages (Figure 7c) being sequestrated by viral particles diminishing toxic impact and ROS generation. Therefore, bacteria can grow in the presence of Cu 10 mM as the supplementation of phages KTN4 or LUZ19 dramatically reduces the number of free cupric ions. Cu^2+^ may also bind to culture media components used for bacterial cultivation lowering the effective ion concentration. In that context, both media and phage particles could serve as binding sites for cupric ions in a saturation effect protecting bacteria from copper toxic activity (Figure 7c). Taking the above into consideration, the supplementation of phage treatment with metal ions might result in an adverse effect on viral particle infectivity as well as in the reduction of the toxic antibacterial concentration of metal ions. Our study showed that phages differed in the sensitivity to copper toxic activity and the ability to sequestrate cupric ions, likely due to the composition of virion proteins, suggesting that each combination should be first examined in detail.

**Figure 8:**
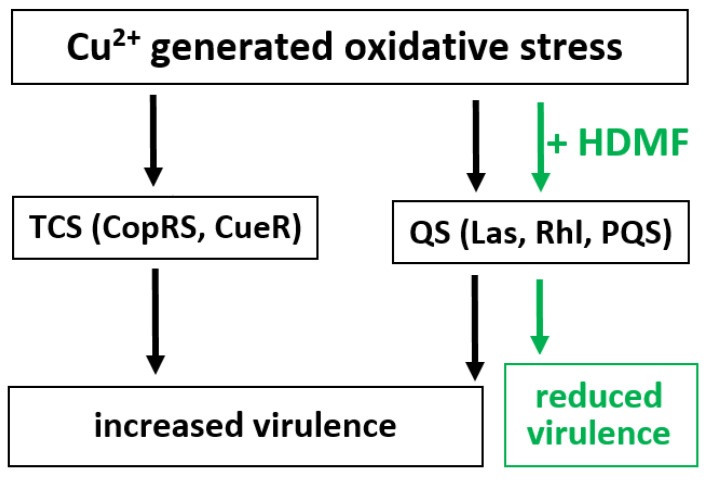
The proposed model of anti-virulent action of HDMF in combination with Cu^2+^. The cupric ions generated stress induces the neutralizing mechanisms regulated by TCS and QS systems, leading to increased virulence of the bacterium. In contrast, HDMF interferes with QS molecules reducing the expression of virulence determinants.

Our second main question was to test whether the selected combinations influence the virulence of *P. aeruginosa* PAO1. Surprisingly, HDMF alone did not reduce virulence determinant production displayed as the RVF values. Our results are contrary to those presented by Choi et al. who obtained reduced biofilm formation (42.6%), as well as LasA protease (53.8%), rhamnolipid (40.9%), and pyocyanin (51.4%) production in the presence of HDMF [16]. We obtained slightly reduced production of elastase (RVF = 0.9) and pyocyanin (RVF = 0.7) but not pyoverdine (RVF = 1), and the growth rate of the population treated with HDMF 10 µM was the same as in the control sample. Presumably, mechanisms responsible for stress-dependent responses were not stimulated, with QS systems impaired by HDMF. Conversely, bacterial treatment with the Cu^2+^ and GE, imposes oxidative and selective stress, respectively, forcing cells to activate protection mechanisms [19,30,59,60,61]. Among them, copper export, sequestration by different molecules, and oxidase production are the most important [59,61]. The complex and multifactorial control of those mechanisms is mainly maintained by QS and TCS systems (Figure 8), further impacting bacterial virulence [25,30]. Cells immediately respond to Cu^2+^ presence by slowing down the metabolism, although, the growth can be restored once the steady-state is reached [60]. Our experimental growth curve for sub-MIC Cu 5 mM showed a similar pattern of reduced final OD_600_ value. Moreover, a high excess in elastase and pyocyanin amount was detected (RVF calculations) proving strong up-regulation of QS-dependent genes. Such effect was not observed for pyoverdine synthesized mainly in response to iron depletion [37]. The *Pseudomonas* possesses specific mechanisms to deal with cupric ion toxicity such as efflux systems, a putative copper-binding periplasmic chaperone, cytoplasmic CopZ proteins, as well as CueR sensor and cytoplasmic CopR/S regulon [60]. Our study confirmed that in the presence of Cu 5 mM (sub-MIC), the virulence factors were heavily overproduced according to the untreated control. The combination of Cu 5 mM with HDMF gave a significant reduction in the RVF values, especially for QS-regulated elastase and pyocyanin. Although this combination showed a worse antibacterial effect, the reduction of virulence determinant production per single cell unit was tremendous. The anti-virulent effect was likely the result of previously described HDMF competition with QS molecules responsible for the regulation of elastase and pyocyanin production dependent on Las and Rhl QS systems [25,33]. Our data confirmed the anti-virulent activity of HDMF, but only when combined with the stressor agents (Figure 8).

GE, at tested sub-MIC concentration and its combination with HDMF, did not significantly impair virulence determinant production expressed as RVF. The strongest impact was observed for ribosomal-dependent elastase production and pyocyanin, whereas the weakest effect was seen for ribosomal-independent pyoverdine production. Therefore, we proved that GE and the GE–HDMF combination are potent in lowering the overall *P. aeruginosa* virulence.

**Figure 9:**
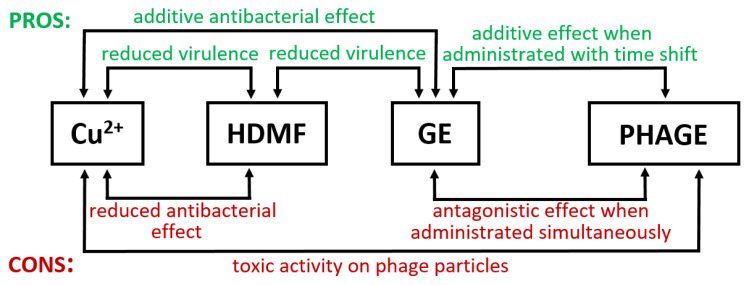
The pros and cons of Cu^2+^, HDMF, GE, and lytic phages combinations.

The outcomes of this study indicate that both pros and cons can be observed for different antibacterial combinations (Figure 9).

The best additive effect was obtained for the Cu^2+^–GE mixture. The combination of GE with phages can be beneficial but not when administrated simultaneously. HDMF applied with Cu^2+^ or GE reduces the overall virulence potential of *P. aeruginosa* PAO1. Furthermore, HDMF neutralizes cupric ion toxicity on the cell-viability level. To conclude, the design of various antibacterial compositions should be accompanied by thorough analyses and critical investigation in terms of the mode of action of each component/agent to exclude mutually adverse interactions.

## 4. Materials and Methods

### 4.1. Bacterial Strain and Phages

The *Pseudomonas aeruginosa* PAO1 (ATCC 15692) reference strain was used. Bacteria were stored at −70 °C in Trypticase Soy Broth (TSB, Becton Dickinson and Company, Cockeysville, MD, USA) supplemented with 20% glycerol. *Pseudomonas* phages KT28, KTN4, and LUZ19 were propagated as previously described [62]. The phage titre was assessed using the double-agar layer technique. Phage culture purity was also confirmed genetically using specific primers, listed in Table S3, to exclude potential contamination. Purified phage samples were stored at 4 °C. Phage characteristics are presented in Table 3.

### 4.2. Antibacterial Activity Assays of Tested Chemicals

Copper(II) nitrate hemi (pentahydrate) (Cu(NO_3_)_2_ × 2.5 H_2_O) (Cu), 4-Hydroxy-2,5-dimethyl-3(2H)-furanone (HDMF), and gentamicin (GE) were obtained from Sigma-Aldrich Chemie GmbH (Steinheim, Germany).

Minimal Inhibitory Concentration (MIC) of HDMF, Cu, and GE were performed by a broth microdilution method according to the EUCAST standards with the modification of a TSB medium used instead of the standard Muller Hinton Broth (MHB) (https://www.eucast.org; Version 2.0; accessed on 2 March 2020). HDMF was tested at 1, 10, and 100 µM; GE at 0.25, 0.5, 1, and 2 µg/mL; Cu(NO_3_)_2_ at 2.5, 5, 10, and 25 mM. The 18-h culture on Trypticase Soy Agar (TSA, Oxoid, Basingstoke, UK) was suspended in saline with an optical density of 0.5 McF (~5 × 10^8^ CFU/mL) and diluted to establish the starting CFU/mL = 10^6^ of each sample. Single agents (HDMF, Cu, GE) or agent combinations (HDMF + GE, HDMF + Cu, Cu + GE) at all aforementioned concentrations were prepared according to the checkerboard assay procedure [65]. One ml of each sample was transferred to the 24-well titration plate and incubated for 20 h at 37 °C with agitation. The absorbance measurement (λ = 600 nm) and CFU/mL enumeration were performed after incubation using a microplate reader (Varioskan Lux, Thermo Scientific, Vantaa, Finland) and standard dilution technique, respectively. Fractional inhibitory concentration index (FIC_I_) for a combination of cupric ions and GE was calculated according to the method described elsewhere [65]. The experiment was performed at least in triplicate. For further experiments, HDMF 10 µM, GE 1 µg/mL, Cu 5, and 10 mM have been chosen.

### 4.3. Anti-Virulent Activity of Tested Agents

For anti-virulent activity testing, an overnight bacterial culture was diluted to CFU/mL = 10^6^ and each antibacterial sample was prepared as described in Section 4.2. Single agents (HDMF 10 µM, Cu 5 mM, Cu 10 mM, GE 0.5 µg/mL, GE 1 µg/mL) or agent combinations (HDMF 10 µM + GE 0.5 µg/mL, HDMF 10 µM + GE 1 µg/mL, HDMF 10 µM + Cu 5 mM, HDMF 10 µM + Cu 10 mM, Cu 5 Mm + GE 0.5 µg/mL, Cu 5 mM + GE 1 µg/mL, Cu 10 mM + GE 0.5 µg/mL, Cu 10 mM + GE 1 µg/mL) were prepared. Plates were incubated for 20 h at 37 °C with agitation. To investigate the level of pyocyanin production the supernatant absorbance was measured at λ = 695 nm (Varioscan Lux, Thermo Scientific) [66]. For pyoverdine production, the fluorescence of the supernatant was measured at λ_ex_ = 392 nm and λ_em_ = 460 nm (Varioscan Lux, Thermo Scientific) [67]. For elastase production, the culture supernatant was mixed with 100 mM Tris-HCl, 1 mM CaCl_2_ buffer (pH 7.5), and 5 mg Elastin-Congo Red (Sigma-Aldrich Chemie GmbH Steinheim, Germany). Samples were incubated with agitation at 37 °C for 3 h. The reaction was stopped by 15 min incubation on ice. Finally, the absorbance was measured at λ = 495 nm in a microplate reader (Varioscan Lux, Thermo Scientific) [68].

To compare the production of virulence determinants per cell unit (Equation (1)), obtained quantifications of virulence determinants (pyocyanin, pyoverdine, elastase) were divided by a sample CFU/mL and presented as a relative virulence factor production (RVF) per cell unit compared to the control (RVF_control_ = 1).

(1) $$\;\mathrm{sample}\;\mathrm{RVF}\;=\;\frac{\frac{\mathrm{sample}\;\mathrm{qVF}}{\mathrm{sample}\;\mathrm{CFU}/\mathrm{mL}\;}}{\frac{\mathrm{control}\;\mathrm{qVF}}{\mathrm{control}\;\mathrm{CFU}/\mathrm{mL}}}$$

RVF—relative virulence factor production per cell unit;
qFV—quantification of virulence factor.

### 4.4. Growth of P. aeruginosa PAO1 in the Presence of Tested Agents Alone or with Phages

The growth of *P. aeruginosa* PAO1 culture was estimated by measuring the optical density (OD_600_) with a starting CFU/mL = 10^6^ of each sample prepared as described in Section 4.2. The bacterial culture was infected with phages KT28, KTN4, or LUZ19 at MOI = 10. The growth kinetics were monitored in a microplate reader (Varioscan Lux, Thermo Scientific) when incubated for 20 h at 37 °C with agitation. The absorbance measurement (λ = 600 nm) was performed automatically every 20 min. Each assay was repeated at least in triplicate. After incubation, additional visual inspection of bacterial growth rate and the medium was carried out, and photographs were taken.

### 4.5. Antibiotic Sensitivity Patterns

Antibacterial susceptibility testing was performed by the disc diffusion test according to EUCAST recommendations (https://www.eucast.org/ast_of_bacteria; Version 8.0; accessed on 1 January 2020). For antibacterial susceptibility testing, the following antibiotics were used: cefotaxime (CTX) 30 µg, ceftazidime (CAZ) 30 µg, piperacillin (PIP) 100 µg, piperacillin/tazobactam (TZP) 100/10 µg, imipenem (IPM) 10 µg, gentamicin (GE) 10 µg, amikacin (AK) 30 µg, ciprofloxacin (CIP) 5 µg (Becton Dickinson and Company, Cockeysville, MD, USA). The bacteria were incubated for 20 h at 37 °C with agents and their combinations as described in Section 4.3. and diluted in saline to the optical density equal to the McFarland No. 0.5. (Densimat, BioMerieux). Cultures were then inoculated on a cation-adjusted Mueller Hinton II Agar plate (MHA) (Becton Dickinson and Company, Cockeysville, MD, USA) with antibiotic discs placed on the lawn. The plates were incubated at 35 °C for 18–20 h before being examined for the zones of growth inhibition according to EUCAST interpretation tables (https://www.eucast.org/ Version 8.0, accessed on 1 January 2020).

### 4.6. Phage Propagation in the Presence of Different Agents

The bacterial culture was prepared as described in Section 4.2 with a starting CFU/mL = 10^6^ of each sample. Phages KT28, KTN4, and LUZ19 were added to the wells to a final titre of 10^7^ PFU/mL (MOI = 10). Plates were incubated with agitation at 37 °C for 20 h. Before and after incubation, the PFU/mL was enumerated using the spot method on a TSA medium (the double-agar layer technique).

### 4.7. Phage Particles Inactivation by Different Agents

The single agents and combinations (without bacteria) were prepared as described in Section 4.3. Phages KT28, KTN4, and LUZ19 were added to the wells to a final titre of 10^7^ PFU/mL. Plates were incubated with agitation at 37 °C for 4 h. Before and after incubation, the PFU/mL was enumerated using the spot method on a TSA medium (the double-agar layer technique).

### 4.8. Copper Ions Impact on Phage Particles Morphology

Phages KT28, KTN4, and LUZ19 (10^8^ PFU/mL) were suspended in a 10 mM Cu solution in saline and incubated with agitation at 37 °C for 4 h. As controls, phages suspended in saline were used. All samples were dialyzed against saline for 24 h in Float-A-Lyzers (Spectra/Por Float-A-Lyzers G2, Spectrum Laboratories, Inc., Rancho Dominguez, CA, USA). Phage titre was determined after dialysis using the spot method as described above. Additionally, after incubation, samples were centrifuged at 25,000× *g* for 60 min. The pellet was washed twice in ammonium acetate (0.1 M, pH 7.0). Phage particles were deposited on copper grids with carbon-coated Formvar films (Sigma-Aldrich Co., St. Louis, MO, USA), stained with uranyl acetate (2%, pH 4.5), and examined under the TEM (Carl Zeiss AG–Zeiss EM900 Transmission Electron Microscope; Jena, Germany) microscope for qualitative changes to verify the effect of copper ions on phage capsids.

### 4.9. Statistical Analysis

The data were statistically elaborated using one-way-ANOVA and the Levene test, followed by the Tukey test. All of the calculations were carried out using the Origin Pro 8.5 software package (OriginLab Corporation). Values of *p* < 0.05 were considered significantly different.

## Figures and Tables

**Table 1:** Phage PFU/mL variation in tenfold change (∆log PFU/mL) after 20 h propagation on bacterial culture supplemented with tested agents.

Antibacterial/Anti-Virulent Agents	∆log PFU/mL (Initial 10^7^ PFU/mL)		
	Phage KT28	Phage KTN4	Phage LUZ19
control	+2	+1	+3
HDMF 10 µM	+2	+1	+3
GE 1 µg/mL	-	-	+1
Cu 5 mM	+2	+1	+3
Cu 10 mM	−2	−5	−5

- no fold change of PFU/mL.

**Table 2:** Phage infective particles enumeration in tenfold change (∆log PFU/mL) after 4 h incubation with selected agents.

	Antibacterial/Anti-Virulent Agents	PFU/mL Reduction of the Initial 10^7^ [∆log]		
		Phage KT28	Phage KTN4	Phage LUZ19
Neutral agents	HDMF 10 µM	-	-	-
	GE 1 µg/mL	-	-	-
	HDMF 10 µM + GE 1 µg/mL	-	-	-
Reducing agents	Cu 5 mM	-	−2	−2
	HDMF 10 µM + Cu 5 mM	-	−2	−2
	Cu 5 mM + GE 1 µg/mL	-	−2	−2
	Cu 10 mM	−2	−5	−5
	HDMF 10 µM + Cu 10 mM	−2	−5	−5
	Cu 10 mM + GE 1 µg/mL	−2	−5	−5

- no fold change of PFU/mL.

**Table 3:** Characteristics of phages used in the study.

Phage	Taxonomy (Family, Genus)	Genome Size [bp]	GenBank Accession Number	Recognized Bacterial Receptor	References
KT28 *	*Myoviridae Pbunavirus*	66,381	KP340287	LPS	[62]
KTN4 *	*Myoviridae Phikzvirus*	279,593	KU521356	T4P	[63]
LUZ19 **	*Autographiviridae, Phikmvvirus*	43,548	NC_010326	T4P	[64]

LPS-lipopolysaccharide, T4P-type IV pili; * from the collection of the Department of Pathogen Biology and Immunology, Institute of Genetics and Microbiology, University of Wroclaw, Wroclaw, Poland; ** from the collection of the Laboratory of Gene Technology, KU Leuven, Leuven, Belgium.

## Data Availability

The data presented in this study are available on request from the corresponding author.

## Supplementary Materials

The following are available online at https://www.mdpi.com/article/10.3390/ijms22189830/s1.

## Abbreviations

HDMF	4-hydroxy-2,5-dimethyl-3(2H)-furanone
GE	Gentamicin
Cu	Copper (II) nitrate hemi (pentahydrate)
CFU	Colony Forming Units
PFU	Plaque Forming Units
MIC	Minimum Inhibitory Concentration
MOI	Multiplicity of Infection
