# Supplementary material for: The In Vitro Anti-Pseudomonal Activity of Cu2+, Strawberry Furanone, Gentamicin, and Lytic Phages Alone and in Combination: Pros and Cons
Source: Int J Mol Sci. 2021 Sep 11;22(18):9830. doi: 10.3390/ijms22189830 (PMC8469652; doi:10.3390/ijms22189830)
Supplement: Supplementary file 1 [file ijms-22-09830-s001.zip › ijms-1285245-SI.pdf]

The *in vitro* anti-pseudomonal activity of Cu^{2+}, strawberry furanone, gentamicin, and lytic phages alone and in combination: pros and cons

Agata Dorotkiewicz-Jach [1,*], Pawel Markwitz [1] and Zuzanna Drulis-Kawa [1,*]

Department of Pathogen Biology and Immunology, Institute of Genetics and Microbiology, University of Wroclaw, Przybyszewskiego 63/77, 51-148 Wroclaw, Poland

* Correspondence: zuzanna.drulis-kawa@uwr.edu.pl, Tel.: (+48) 71 375 6290;

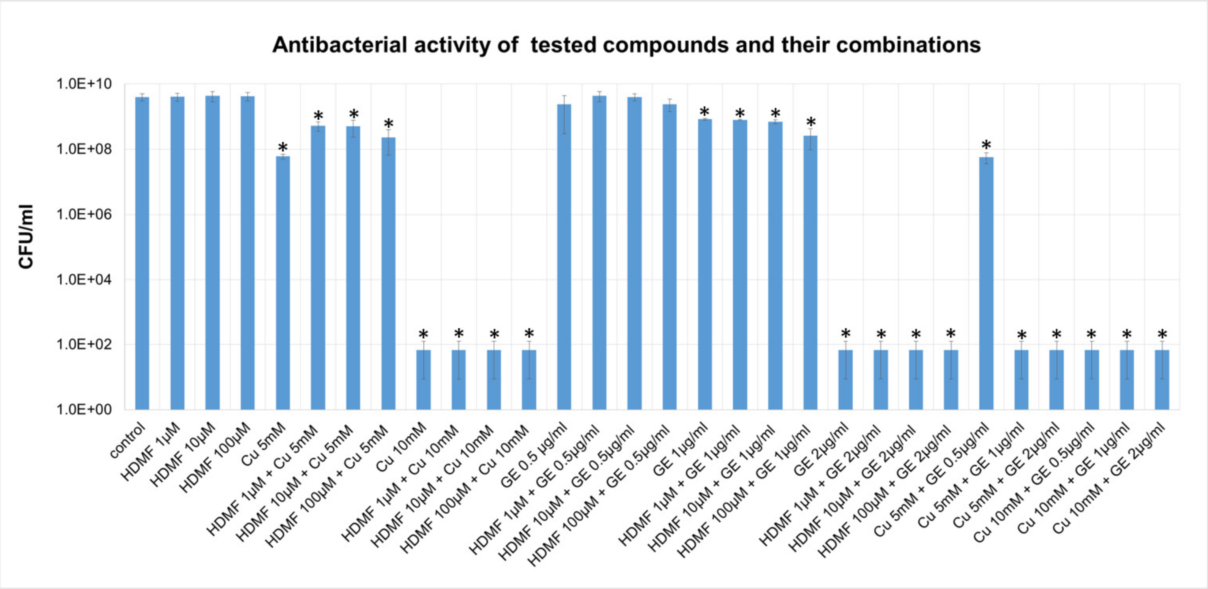

Figure S1. The impact of Cu^{2+}, HDMF, and GE on 20 h growth of *P. aeruginosa* PAO1; *statistically significant differences according to PAO1 untreated control ($P<0.05$)

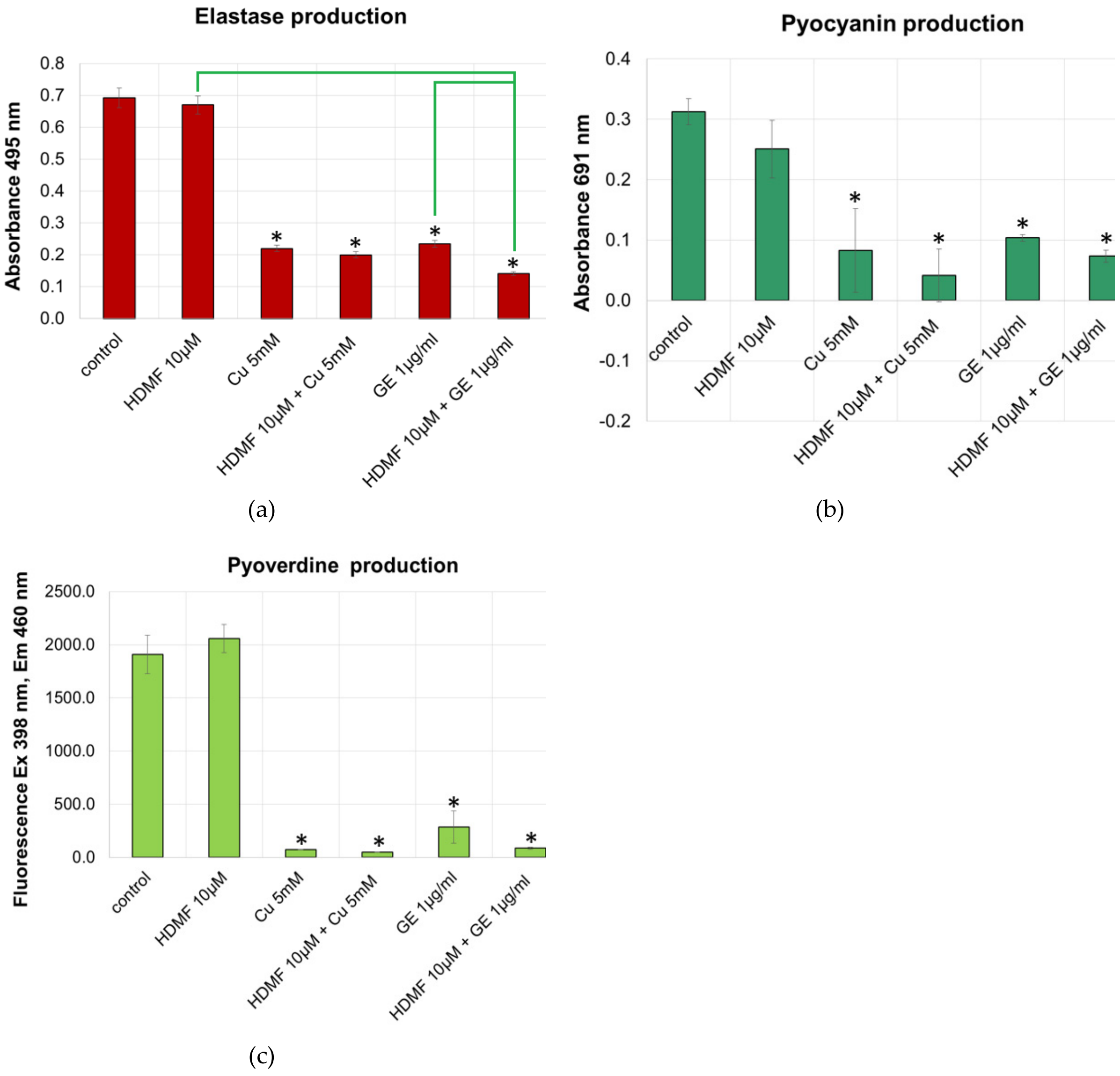

Figure S2. The impact of Cu^{2+}, HDMF, and GE on *P. aeruginosa* PAO1 virulence determinants production: (**a**) elastase production; (**b**) pyocyanin production; (**c**) pyoverdine production; *statistically significant differences according to PAO1 control ($P<0.05$), brackets indicate statistically significant differences between agent combinations ($P<0.05$).

Table S1. Statistical analysis of the significance of differences between tested combinations of antibacterial and anti-virulent components (P<0.05).

	Figure 1b		Figure 1c		Figure 1d	
	HDMF 10μM GE 1μg/ml	HDMF 10μM Cu 5mM	HDMF 10μM GE 1μg/ml	HDMF 10μM Cu 5mM	HDMF 10μM GE 1μg/ml	HDMF 10μM Cu 5mM
HDMF 10μM	Y	Y	Y	Y	Y	Y
Cu 5mM		N		N		N
GE 1μg/ml	Y		N		N	

Y-statistically significant with P<0.05; N-statistically not significant;

Table S2. The impact of Cu^{2+}, HDMF and GE alone and in combination on antibiotic sensitivity patterns of *P. aeruginosa* PAO1.

Tested antibiotic	PAO1 (control)	HDMF 10 μM	Cu 5 mM	HDMF 10μM Cu 5mM	GE 0,5 μg/ml	HDMF 10μM GE 0,5μg/ml	Cu 5mM GE 0,5μg/ml
CTX	24-25^{S}	-	-	-	-	-	-
CAZ	29-31^{S}	-	-	-	-	-	-
PIP	31-32^{S}	-	-	-	-	-	-
TZP	33-35^{S}	-	-	-	-	-	-
IPM	27-29^{S}	-	-	-	-	-	-
CN	18-19^{S}	-	-	-	-	-	-
AK	24-25^{S}	-	-	-	-	-	-
CIP	36-38^{S}	-	-	-	-	-	-

Numbers indicate diameters of zones of inhibition in mm, S-sensitive according to EUCAST recommendations (https://www.eucast.org/ast_of_bacteria/), – no change observed,

Table S3. The starters used for genetic confirmation of phage culture purity.

Phage	Genome size	GenBank accession number	Starter sequence	Melting temperature [°C]
KT28	66,381 bp	KP340287	(F) CTTCGACGCCGACCGGGAATC<br>(R) GATATGCTACGCCGCCCATTGTG	60.2<br>58.8
KTN4	279,593 bp	KU521356	(F) CTAACTAAATATATTGACGCTGTATCG<br>(R) CCGCTATATACTTGTAGATAACGC	53.7<br>54
LUZ19	43,548 bp	NC_010326	(F) CTAACTAAATATATTGACGCTGTATCG<br>(R) CAACCTCCAGCCAATCTCAATAAAAATT	62<br>57.3
